# Nasal cytology in children: recent advances

**DOI:** 10.1186/1824-7288-38-51

**Published:** 2012-09-25

**Authors:** Gelardi Matteo, Marseglia Gian Luigi, Licari Amelia, Landi Massimo, Dell’Albani Ilaria, Incorvaia Cristoforo, Frati Franco, Quaranta Nicola

**Affiliations:** 1 Department of Otolaryngology, University of Bari, Bari, Italy; 2 Department of Pediatrics, Foundation IRCCS Policlinico San Matteo, University of Pavia, Italy, P.le Golgi, 2-27100, Pavia (PV), Italy; 3 Paediatrics, ASL TO1 Turin, Turin, Italy; 4 Medical and Scientific Department, Stallergenes, Milan, Italy; 5 Allergy/Pulmonary rehabilitation, ICP Hospital, Milan, Italy

**Keywords:** Nasal cytology, Allergic rhinitis, Non-allergic rhinitis, Classification, Allergen immunotherapy

## Abstract

Nasal cytology is a very useful diagnostic tool in nasal disorders, being able to detect both the cellular modifications of the nasal epithelium caused by either allergen exposure or irritative stimuli (that may be physical or chemical, acute or chronic), or inflammation. Over these past few years, nasal cytology has allowed to identify new disorders, such as the non-allergic rhinitis with eosinophils (NARES), the non-allergic rhinitis with mast cells (NARMA), the non-allergic rhinitis with neutrophils (NARNE), and the non-allergic rhinitis with eosinophils and mast cells (NARESMA). The rhinocytogram is actually able to distinguish the different forms of allergic rhinitis and to suggest the appropriate treatment, such as antinflammatory drugs or allergen immunotherapy. The technique is easy to perform and nasal cytology is therefore particularly suitable even for children. Such a consideration suggests the utility of a systematic use of nasal cytology in the diagnostic work-up of nasal disorders in children, in order to reach a proper defined diagnosis and to set a rational therapeutic approach: in facts, these two elements are fundamental in order to prevent from complications and to improve the patient’s quality of life.

## Review

Nasal cytology is a very useful diagnostic tool in diagnosing nasal allergic disorders [1,2]. The technique allows clinicians to detect the cellular modifications of the nasal epithelium caused by exposure to either physical or chemical [3,4], acute or chronic irritations. Also, it makes it easy to evaluate the different types of inflammation (viral, bacterial, fungal or parasitical) [5,6]. Over the past few years, nasal cytology has shown to be quite an attractive tool in clinical and scientific applications. Indeed, a large number of papers has been published on the cytological characterization of nasal pathologies, and particularly on allergic and non allergic rhinitis. These researches contributed to the understanding of some pathophysiological mechanisms of allergic rhinitis, and to the identification of new disorders, namely the non-allergic rhinitis with eosinophils (NARES), the non-allergic rhinitis with mast cells (NARMA), the non-allergic rhinitis with neutrophils (NARNE), and the non-allergic rhinitis with eosinophils and mast cells (NARESMA) [7-9].

### The cytological aspects of nasal mucosa and microscopic techniques

**Figure 1:**
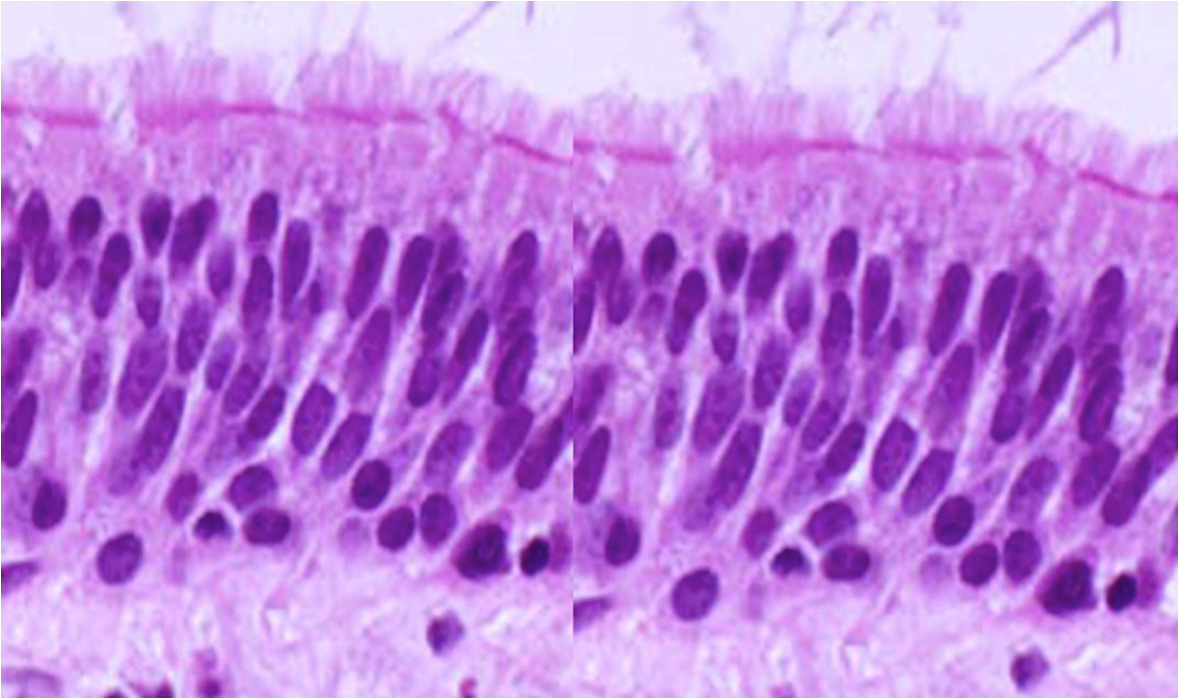
**Nasal Mucosa: the ciliated pseudo-stratified epithelium, composed of ciliated mucous-secreting cells (striated and basal).** Staining MGG 400X.

**Figure 2:**
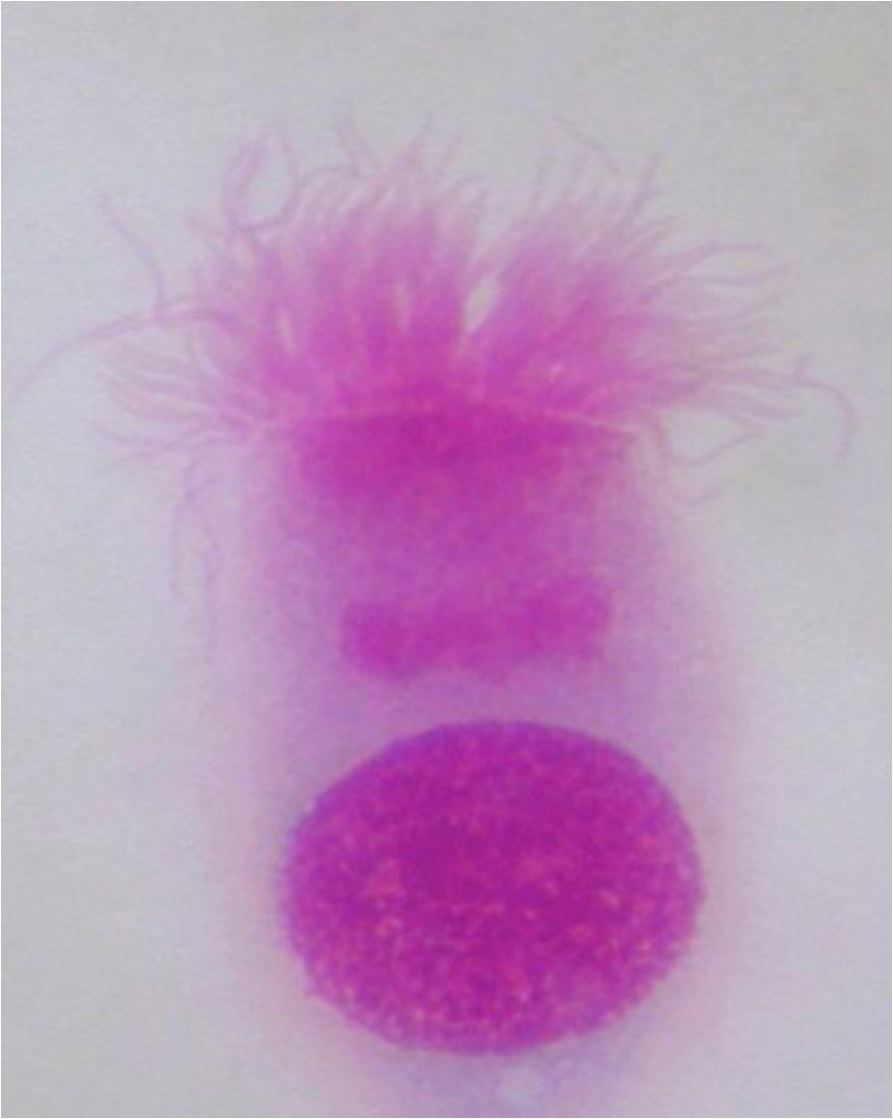
**Nasal Mucosa: the ciliated cell, part of the mucus-ciliated system, representing an important defence localized in the airways.** Staining MGG 2000X.

**Figure 3:**
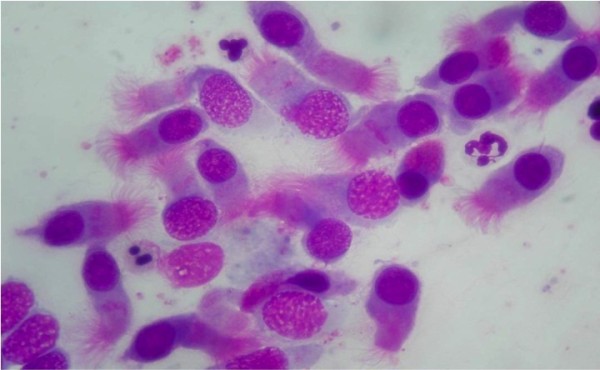
**Normal rhinocytogram: in healthy subjects, the nasal mucosa is composed of numerous ciliated cells that characterize the pseudo-stratified epithelium, and few neutrophils.** Staining MGG 1000X.

The nasal mucosa is formed by a ciliated pseudo-stratified epithelium (Figure 1), which is composed of ciliated mucous-secreting cells, striated and basal. The ciliated cell (Figure 2) is the most differentiated element of the nasal mucosa [10] and, together with mucous-secreting cells, it represents the first-line defence located in the airways (the so-called mucus-ciliated system). The diagnosis of nasal disorders through nasal cytology is based on the consideration that, in healthy subjects, the nasal mucosa is composed of four normal subsets of cells, which commonly characterize the pseudo-stratified epithelium; besides neutrophils, no other cells are detected in healthy individuals (Figure 3). Therefore, on a rhinocytogram, the presence of eosinophils, mast cells, bacteria, spores and fungi has to be considered as a clear sign of nasal pathology.

Nasal cytology was introduced in 1889, when Gollash highlighted the presence of numerous eosinophils in the nasal secretions of an asthmatic patient and suggested that these cells could be the key elements for the pathogenesis of the disease [11]. Eyermann, in 1927, detected the presence of granulocyte eosinophils in the nasal secretions of allergic patients and showed their importance in diagnosing the disease [12]. Thanks to this discovery, a great value was attributed to the identification of specific cellular subsets related to different nasal pathologies [13-15], and this consideration opened the way to the routine use of nasal cytology in the study of allergic and non allergic, infectious and inflammatory rhinitis. Different factors have been responsible for the increased interest for this diagnostic tool and its widespread use: on one hand, the fact that the technique is easy to perform, and, on the other hand, that it is a non-invasive approach. Therefore, this tool may be easily repeated on the same patient, with is essential both in the follow-up of the disease and to monitor the efficacy of medical and surgical interventions. Based on the fact that this method is simple, safe, non-invasive and poorly expensive, it could be routinely used in outpatient clinics at all ages, even in children [16].

The following steps characterize the cytological technique: sampling, processing (with fixing and staining), and observation through microscopy. The cytological sampling consists of collecting the nasal mucosa surface cells and it can be performed either through the use of a sterile swab (such as an oro-pharyngeal swab) or a small scraper made of disposable plastic such as the Rhino-probe (Arlington Scientific, Springville, UT, USA) [17]. Sampling collection may be even done by scraping the middle portion of the inferior turbinate, where there is an optimal ratio between ciliated and mucous-secreting cells, usually in favour of ciliated cells. Nevertheless, on a routine basis, nasal swab is preferred to scraping, since it is easier and less troublesome, using the latter only when investigating more collaborative patients. The sampling step must be carefully performed through anterior rhinoscopy, using a nasal speculum and good lighting. As mentioned before, it is a minimally invasive method, so that local anaesthesia is usually not required.

**Figure 4:**
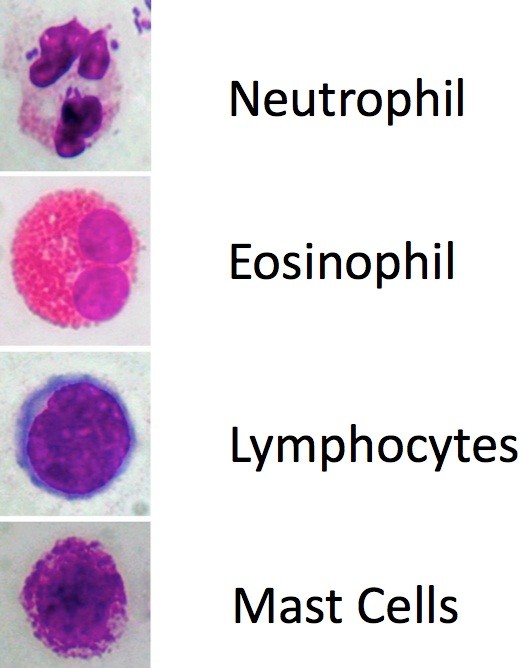
**Immunoflogosis: staining with May-Grunwald-Giemsa (MGG) method, allows to detect all the cellular components of the nasal mucosa, including inflammatory cells, such as neutrophils, eosinophils, lymphocytes and mast cells.** Staining MGG.

When the sampling is obtained, the material is placed on a glass slide, fixed by air drying and stained by May-Grunwald-Giemsa (MGG) method, which allows the detection of all the cellular components of the nasal mucosa, including those cells that are associated to the immune inflammation process (such as neutrophils, eosinophils, lymphocytes and mast cells) (Figure 4), and bacteria, spores and fungi. Staining usually requires about 30 min; nevertheless, new staining systems are currently available (MGG QUICK STAIN Bio-Optica, Milan, Italy), allowing completing such step in less time.

The slide is then observed through a light microscopy supplied with an object-glass, able to magnify up to 1,000x. For the rhinocytogram analysis, at least 50 microscopic fields have to be read in order to detect eosinophils, mast cells, neutrophils, bacteria, spores, calculating their percentages and reach a diagnosis [16,17].

### Nasal cyto-pathology

Several nasal pathologies have been identified and a large number of classifications appear in scientific journals, although a unique classification has not yet been accepted. In Additional file 1: Table S1 we propose a classification that aims to be complete and comprehensive, and involves a heterogeneous range of diseases. From a cellular point of view, nasal pathologies first affect the ciliated cells, determining a rearrangement of the epithelium in favour of mucous-secreting cells (mucous-secreting metaplasia). This process has important pathophysiological and clinical consequences, because the increase of mucous-secreting cells causes a major production of mucous, while the decrease in ciliated cells leads to a reduced efficiency of the muco-ciliated transport. These events favour the stasis of mucous secretions in the nose, determining a major risk of bacterial infection [18]. Considering that the turnover of a ciliated cell takes about three weeks, frequent inflammation does not allow the re-establishment of a normal ratio between the different cellular subsets [19,20].

### Nasal cytology in allergic and non-allergic rhinitis

**Figure 5:**
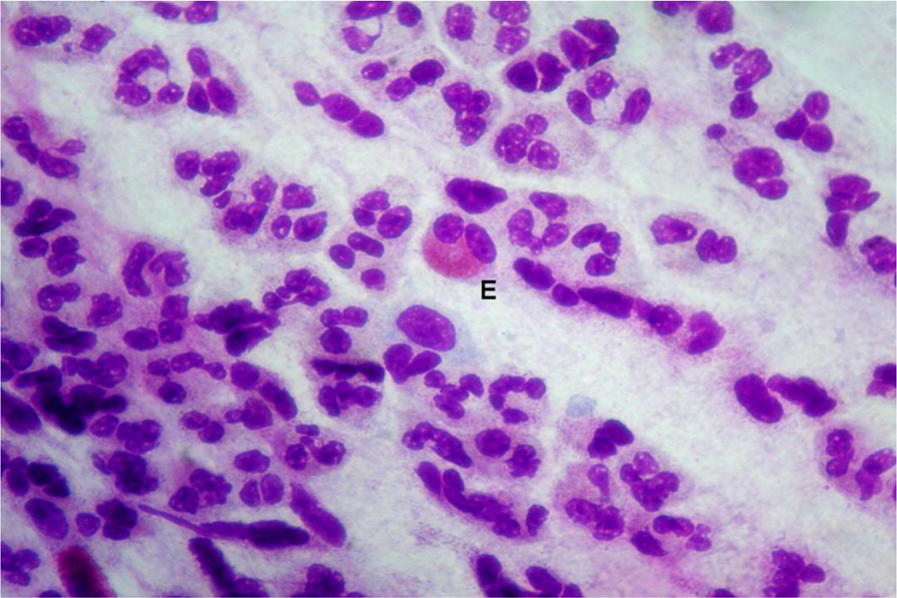
**Perennial allergic rhinitis: if allergen exposure is persistent throughout the year, a minimal persistent flogosis may be detected, characterized by a persistent infiltration of neutrophils and few eosinophils (E).** Staining MGG 1000X.

**Figure 6:**
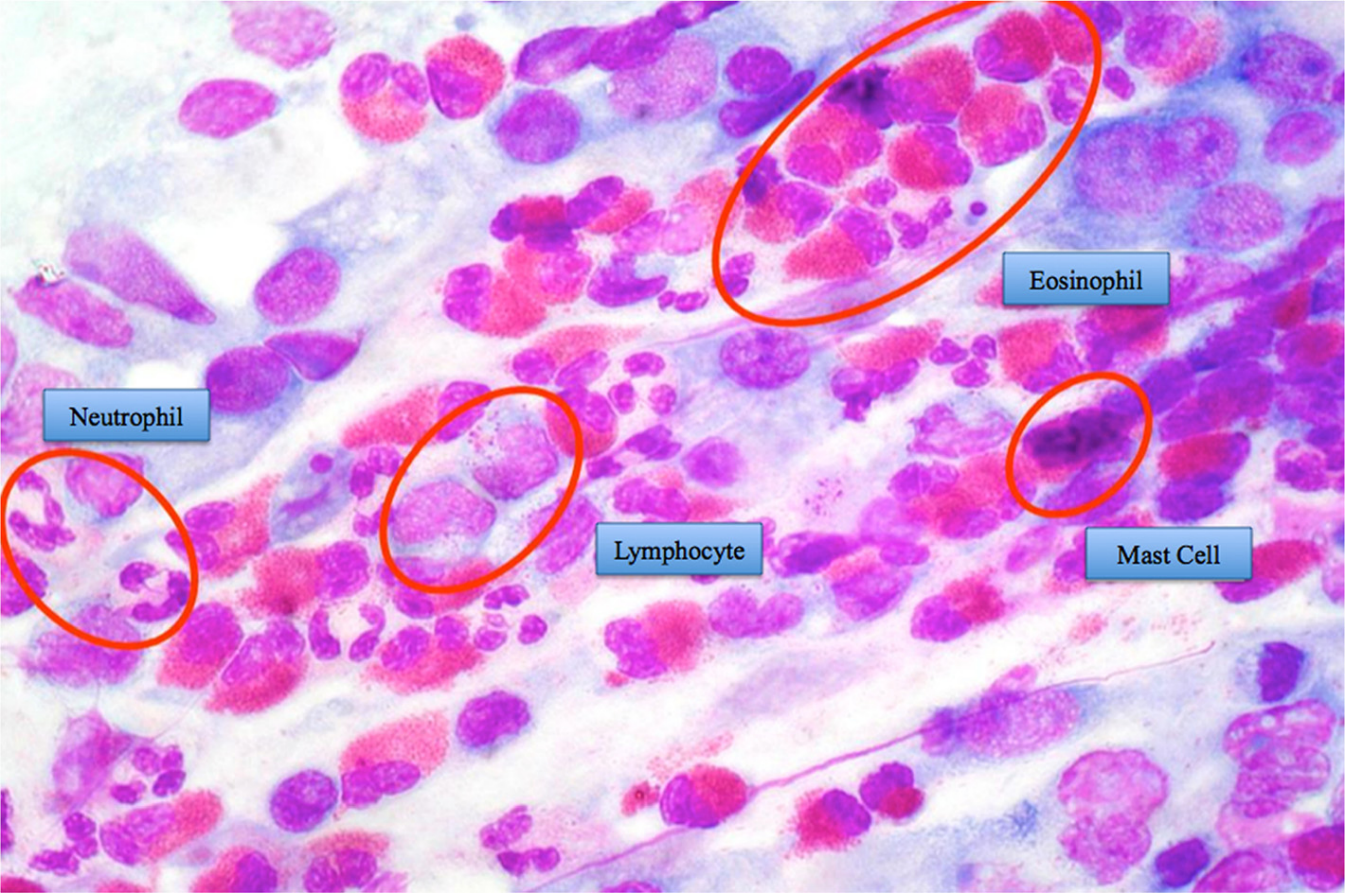
**Seasonal allergic rhinitis: nasal cytology in a patient evaluated during the pollen season; the sample is characterized by the presence of numerous neutrophils, lymphocytes, eosinophils and mast cells, partially degranulating.** Staining MGG 1000X.

**Figure 7:**
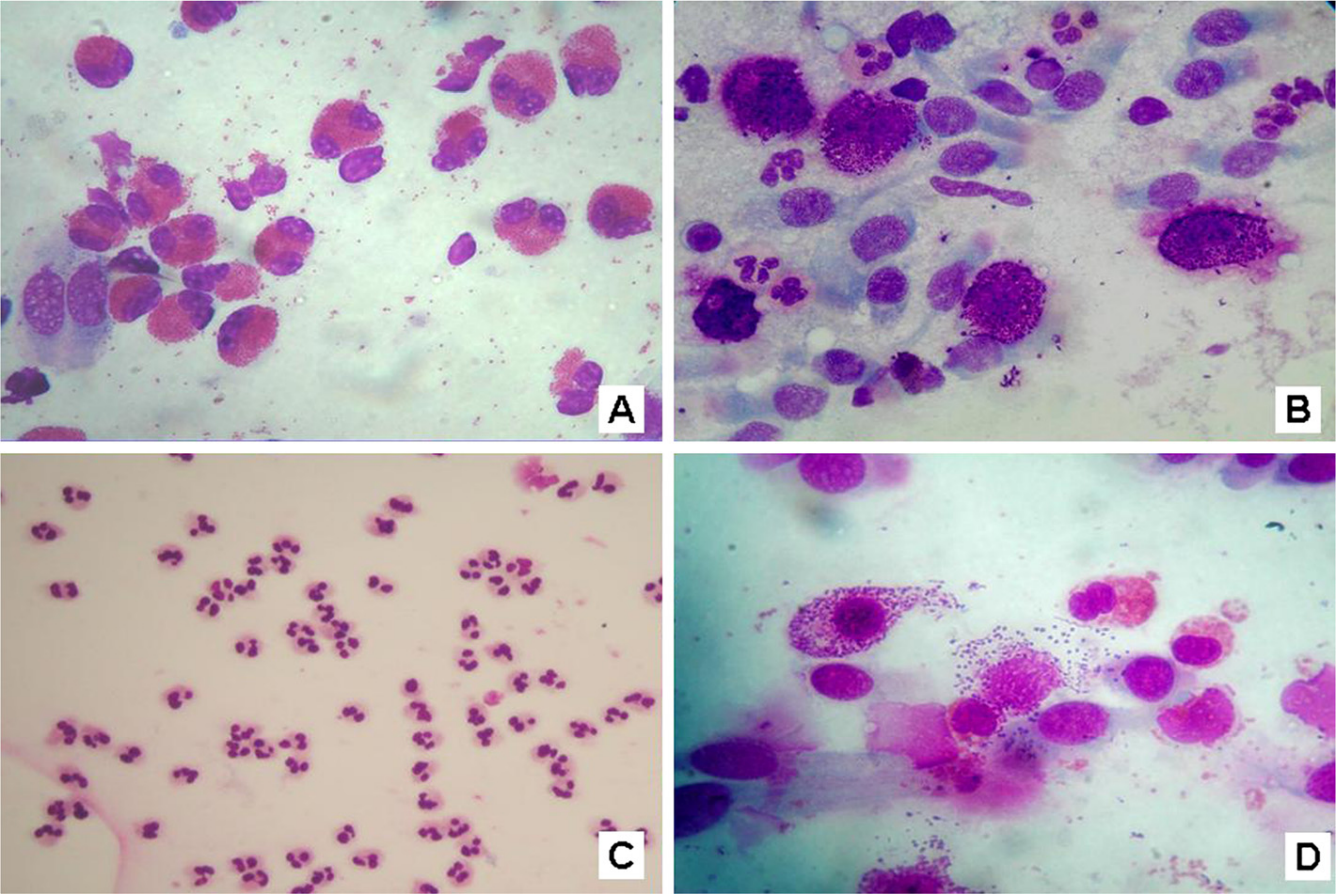
**Non IgE-mediated rhinopathies in children: non-allergic rhinitis with eosinophils (NARES, A), non-allergic rhinitis with mast cells (NARMA, B), non-allergic rhinitis with neutrophils (NARNE, C), non-allergic rhinitis with eosinophils and mast cells (NARESMA, D).** Staining MGG 1000X.

Patients suffering from seasonal or perennial allergic rhinitis (AR), when exposed to the causative allergen, either in the environment or during a nasal provocation test, develop an immediate nasal response, the so-called *early phase*, and then a late phase response [21,22]. From a microscopic point of view, these responses are characterized by a mucosal infiltration of inflammatory cells (eosinophils, mast cells, neutrophils and lymphocytes), which cause the IgE-mediated symptoms (itching, nasal congestion, rhinorrhoea, sneezing) due to the release of the different chemical mediators released by such cells. When the intensity of allergen exposure is low but continual, as in persistent rhinitis (caused, for example, by house dust mites allergy), a “minimal persistent inflammation” occurs, characterized by a persistent infiltration of neutrophils and, only minimally, of eosinophils [23,24] (Figure 5). Mast cells and degranulating eosinophils are rarely found. The above-mentioned cellular condition is clinically translated into a sub-chronic symptomatology, which characterizes the patients suffering from perennial AR. Main symptoms include nasal congestion and rhinorrhea. As for seasonal AR, the rhinocytogram may be quite heterogeneous, depending on the period of the year during which the patient is explored, that is to say during or outside the pollen season. In fact, during the pollen season, patients show all the clinical signs of the disease, and nasal cytology is characterized by the presence of neutrophils, lymphocytes, eosinophils and mast cells, mostly degranulating (Figure 6). By contrast, in patients evaluated outside the pollen season, there are no clinical or cytological signs that may be detected; this aspect is even more evident if the pollen season and the allergen exposure finished more than 30 days before evaluation. In these cases, for an effective diagnosis, it is mandatory to perform a nasal provocation test with the specific allergen or a cytological study during the peak of pollination. A study by our group on patients suffering from AR showed some striking data: subjects with perennial rhinitis and mono-sensitized subjects suffering from pollen-induced AR show different concentrations of inflammatory cells and nasal resistance measured by rhinomanometer, if compared with different types of patients with AR [25]. It is noteworthy that pollen-induced AR is able to induce higher levels of tissue inflammatory cells (eosinophils, neutrophils and mast cells), and of nasal resistance. Besides the differences in terms of cellular subsets, as for eosinophils and mast cells, some modifications have been found in terms of degranulation level, which varies in accordance with the considered pollen (grass, parietaria, cypress and olive). Nasal eosinophilia characterizes allergic diseases at all ages; the presence of intra- and extra-cellular bacteria is the mark of a concomitant bacterial infection (allergic rhino-sinusitis). Investigating the nasal cytology in 1013 children (personal data) aged 0–13 years, we have been able to detect different immunological inflammatory patterns. Moreover, with such a cohort, we highlighted the existence of non IgE-mediated rhinopathies in children: non-allergic rhinitis with eosinophils (NARES, Figure 7A), non-allergic rhinitis with mast cells (NARMA, Figure 7B), non-allergic rhinitis with neutrophils (NARNE, Figure 7C), non-allergic rhinitis with eosinophils and mast cells (NARESMA, Figure 7D). These cellular rhinopathies present a chronic-progressive course, more intense symptoms, and induce local-regional complications (such as rhinosinusitis, and frequent otitis) and respiratory complications (such as bronchitis, pneumonia, asthma, rhino-bronchial syndrome). If these rhinopathies are not effectively controlled by pharmacotherapy, 20 years later they may show an evolution towards nasal polyposis [26].

The NARESMA form, a recently described disorder [8], is the disease with the greatest tendency to develop complications (nasal polyposis and/or asthma), with the worst quality of life and severe sleep disturbances (continuous awakenings, snoring, sleep-apnea syndrome).

### “Overlapped” rhinitis

The most important contribute of nasal cytology to the diagnosis of rhinopathies is the introduction of the so-called *overlap* concept; thanks to the cytological approach, it is actually possible to identify patients simultaneously suffering from more than one form (for example AR associated to NARES, NARESMA, etc.). The possibility to identify these clinical conditions allows avoiding a wrong therapeutical approach [27]. Some patients who present a sensitization to seasonal allergens, may experience a perennial symptomatology, along with a positive cytology for eosinophils and mast cells, even outside the pollen season. In this case, the rhinocytological study is very useful tool, since it is able to identify the concomitance of more diseases in accordance with a differential cytological diagnosis.

These clinical conditions are characterized by a more intense vasomotor symptomatology with a chronic course; if not diagnosed and treated adequately by pharmacological therapy, often based on personalized cycles of nasal corticosteroids, and sometimes systemic corticosteroids, antihistamines, antileukotrienes, they will show some sort of complications (such as turbinate dysfunction, rhino-sinusitis, rhino-bronchial syndrome, rhino-otitis). The clinical-therapeutic implications of these conditions are fundamental both for the ENT and the allergy specialist, but also for paediatricians, since these forms occur since childhood. If these patients are properly treated by allergen immunotherapy (AIT), mainly sublingual immunotherapy (which is more accepted both by children and caregivers) [28], they will benefit from the advantages related to such a treatment (blockage of the so-called allergic march, and clinical efficacy persisting also after the end of the immunotherapy course). These patients must be informed that such a positive outcome occur only if AIT pre-requisites are fulfilled. In particular, adequate allergen dosage must be administered for a sufficient duration, corresponding to 3–5 years [29], and patients must be adequately educated in order to ensure an optimal compliance to the prescribed treatment [30].

### Nasal cytology in children

Despite the favourable characteristics of nasal cytology, only scant data are available on its application in children. In 1988 Sala et al. showed that children with chronic rhinitis showed a decrease of the ciliated component and an increase of goblet cells [31] but no other study was addressed on the paediatric population. In 2007, the role of cytology in the diagnosis of rhinosinusitis in children was reappraised [18], however studies on nasal cytology in children with rhinitis are still lacking. A recent study analyzed the histopathology of chronic rhinosinusitis in children as based on different techniques including nasal biopsies [32], but it is obvious that nasal biopsies are hardly feasible as a routine method to detect the inflammatory cells in the nose, while cytology has optimal characteristics to assess this aspect.

## Conclusions

Taking into account all the above considerations, it is desirable for nasal cytology to be systematically performed in the diagnostic work-up of nasal disorders in the paediatric population, in order to reach a better diagnosis and to set a rational therapeutic approach, which are fundamental elements to prevent the well-known complications and to improve the patients’ quality of life.

## Abbreviations

NARES: Non-allergic rhinitis with eosinophils; NARMA: Non-allergic rhinitis with mast cells NARMA; NARNE: Non-allergic rhinitis with neutrophils; NARESMA: Non-allergic rhinitis with eosinophils and mast cells; MGG: May-Grunwald-Giemsa; AR: Allergic rhinitis; AIT: Allergen immunotherapy.

## Competing interests

Franco Frati is the medical and scientific Director of Stallergenes-Italy. Cristoforo Incorvaia is a scientific Consultyant for Stallergenes-Italy. Ilaria Dell’Albani is marketing officer for Stallergenes-Italy. All other Authors have no financial and non-financial competing interest to declare.

## Authors’ contributions

GM Data analysis. MGL Data analysis. LA Data analysis. LM Data analysis. DAI Data analysis. IC Text writing and editing. FF Text writing and editing. QN Data analysis. All authors read and approved the final manuscript.

## Supplementary Material

Additional file 1 **Table S1.** Classification of rhinopathies. Click here for file
