# Supplementary material for: Nasal cytology in children: recent advances
Source: Ital J Pediatr. 2012 Sep 25;38:51. doi: 10.1186/1824-7288-38-51 (PMC3533990; doi:10.1186/1824-7288-38-51)
Supplement: Additional file 1 — Table S1. Classification of rhinopathies. [file 1824-7288-38-51-S1.doc]

**The classification of rhinopathies**

**R** Acute Viral

Infective Bacterial

Chronic Mycotic

**H**  Inflammatory by physical-chemical-atmospheric agents

**I**  Seasonal Replaced by ARIA classification

Allergic Perennial (intermittent/persistent)

**N** Vasomotor Non-allergic rhinitis with neutrophils (NARNE)

Non Allergic Non-allergic rhinitis with eosinophils (NARES)

Non-allergic rhinitis with mast cells (NARMA)

Non-allergic rhinitis with eosinophils and mast cells (NARESMA)

**O**

Hyperplastic/granulomatous Nasal polyposis, choanal-antrum polyps, Wegener's granulomatosis

**P** Sarcoidosis, Churg-Strauss

Tumor Inverted papilloma, fibromas, chondroma, angioma, carcinoma,

sarcoma

**A** Atrophic Senile or characterized by the chronic of particular rhinitis processes

Abuse of α-adrenergics, β-blockers, cocaine, clonidine, ACE

Iatrogenic inhibitors, oral contraceptives, anti-epileptics, neuroleptics, aspirin and other FANS, calcium antagonists

**T**

Hypothyroidism

Hormonal Pregnancy

**H** Pre-Menstrual

Adrenergic or angiospastic-cholinergic

**I** Gustatory (oronasal syndrome)

Mechanical (nasal septum deviation, foreign bodies, choanal atresia, adenoidism)

**E** Others Decubitus-physical exercise

Occupational (allergic and non allergic)

Psychotic-Emotional-Sexual excitement

**S**  Ciliary dyskinesia-cystic fibrosis-meningoencephalocele.
